# A simple image processing approach for electronic cleansing in computed tomographic colonography

**DOI:** 10.2349/biij.5.3.e28

**Published:** 2009-07-01

**Authors:** S Yamamoto, G Iinuma, M Suzuki, T Tanaka, Y Muramatsu, N Moriyama

**Affiliations:** 1 Research Center for Cancer Prevention and Screening, National Cancer Center Japan; 2 Department of Radiology, National Cancer Center Hospital Japan

**Keywords:** Electronic cleansing, CT colonography

## Abstract

The prevalence of colon cancer has seen strong demand in screening for colorectal neoplasia, and this has drawn considerable attention to the technological advances in Computed Tomographic Colonography (CTC). With the assistance of an oral contrast agent, an imaging technique known as Electronic Cleansing (EC), can affect virtual cleaning of the computed tomography (CT) images, to remove fecal material that is tagged by the agent. Technical problems can arise with electronic cleansing however, when the air lumen causes distortions to the tagged regions which result in partial volume effects.

Combining the simple image arithmetic of an electronic cleansing algorithm, with a vertical motion filter at the fluid level of the bowel, artifacts such as those caused by an air lumen are eliminated. Essentially, the filter becomes a vector for that carries the measurement of vertical motion to neutralise the artifact that is causing partial volume effects. Results demonstrate that despite its simplicity, this technique offers accuracy and is able to successfully maintain the normal intra-colonic structure, while supporting digital leaning of tagged residual material appearing on the colon wall.

## INTRODUCTION

In the developed world, colorectal cancer represents one of the most prevalent cancer that causes death [1-3]. While early detection will greatly improve treatment, the cancer can take many years to develop [4]. Cancer organisations and associations devoted to cancer prevention all over the world endorse guidelines for colorectal screening, yet compliance with these recommendations is low [5].

The reason for this lack of participation is due to the perception an invasive conventional colonoscopy holds for individuals; these require sedation and a rigorous bowel preparation [6]. Also known as Virtual Colonoscopy, computed tomographic colonography (CTC) is a promising alternative to conventional colon cancer screening.

The advantages to be enjoyed with CTC over a conventional colonoscopy are many [7]. CTC is a non-invasive technique requiring no sedation and can be completed in a much shorter period of time. While poor image processing can result from electronic cleansing (EC), the powerful diagnostic ability of CTC when accompanied by stool tagging and electronic cleansing (EC) offers a number of advantages [8-10]. Specifically due to the shorter evaluation time and the lower assessment demands, a larger level of diagnostic confidence is found when EC is practiced.

Numerous mathematical models based on statistical data analysis, and adaptive morphological operations have been applied in a bid to cure the problems EC experiences with artifacts in the form of partial volume effects as a result of the air lumen and tagged regions [11,12]. These processing methods however, are either time consuming, or cannot be relied upon to provide a pattern of a normal anatomical structure.

This paper suggests an algorithm to eliminate these artifacts from clinical practice. A vertical motion filter will be configured to the fluid level in the bowel and combined with simple image arithmetic. The artifact of partial volume defects will be reduced or eliminated totally, as the filter becomes the vector for vertical motion. The processing speed of this technique is rapid, simple, and successfully maintains a normal anatomical structure for the purposes of EC.

This novel method of image processing for EC eliminates the artifacts in partial volume defects caused at the boundary between the air lumen and the regions tagged by the contrast agent.

## MATERIALS AND METHODS

### Bowel preparation scheme and CT data acquisition

Upon receiving approval from the Institutional Ethical Review Board, the authors performed a CTC colon cancer screening on a patient with a colonic abnormality. In order to enhance the intensity of stool and fluid for electronic cleansing, a bowel preparation was performed prior to the examination.

The patient was asked to ingest diluted contrast material at each meal the day prior to that of the procedure. Apart from barium-based contrast agents to affect opacification of the bowel homogenously, additional solutions were also taken by the patient to liquidise and enhance the stool under imaging.

At a concentration of 40% w/v (TAGITOL V; EZ-EM, Westbury, NY), 20ml of barium contrast agent was administered with 800ml water after each meal the day prior. In addition, the patient was given a routine bowel preparation that included 50g of magnesium citrate in 150ml (Magcorol, P., Horii Pharmeceutical Ind., Ltd, Osaka, Japan) after dinner, and two tablets of Sennoside (Novartis Pharma tablets: Novartis Pharma K.K., Tokyo, Japan) were taken at bedtime as a laxative accelerant. Finally, a suppository (LECICARBON SERIA Pharmaceutical Co., Ltd. Tokyo, Japan) was administered to the patient upon waking.

While the patient was in both supine and prone positions, CT colonography was performed after bowel opacification, and also after CO_2_ gas insufflations of the colon had occurred using CO_2_ EFFICIENT Endoscopic Insufflator (E-Z-EM, Westbury, NY).

The CT scan was captured by using a Multi-slice Helical CT scanner (Aquillon 16, Toshiba Medical Systems, Tochigi, Japan) and was performed using the following acquisition protocol:

- 120 kVp
- Variable tube current
- Exposure Control Noise level at SD 20
- 0.5 second gantry rotation
- Helical pitch of HP15
- 1 mm collimation x 16 rows
- 1 mm image reconstruction interval

Electronic cleansing (EC) was completed on software developed using the MATLAB framework (MATLAB 2008b;Mathworks, Natick, MA). The program was installed on a standard PC (CPU ; Intel Core2 Duo T9300, Memory:3.00GB, Graphics Card: GeForce 8600M GT and OS: Windows XP).

### Image processing for electronic colon cleansing

In order to provide a cleansed model for Virtual Colonoscopy, electronic cleansing (EC) is used to identify the colonic material that is revealed by a contrast agent. Technical difficulties arise however, with partial volume defects being caused by the boundary between the air lumen and the tagged regions.

**Figure 1:**
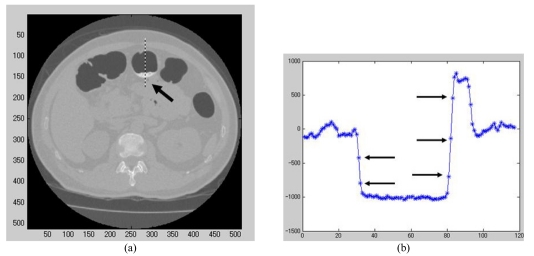
Pixels influenced by partial volume effect. (a) An axial CT image obtained during CTC before EC. Tool tagging was shown in a high intensity part (arrow). (b) Profile plot at the border of air and tagged material. A few pixels influenced by partial volume effect (arrow).

Basically, a template is made of the air material in order to reproduce the partial volume effect of the peripheral edge. Then using the vertical motion filter vector, the vertically spread pixels of the template are de-blurred. The high density tagged materials are substituted with the template of air material using texture synthesis by non-parametric sampling (Efros and Leung’s algorithm [13]). The partial volume effect was reproduced using a simple averaging filter with a kernel size of 3 x 3. The size of the kernel in the averaging filter is governed by the number of pixels that are affected by the partial volume effect (Figure 1).

Using the air material template to reduce the artifact boundary between the air lumen and the tagged regions, the vertical motion filter was applied to the resulting image. The use of the filter reduces the vertical spread of the pixels. The length of the filter kernel is a three row vector array [0.3333, 0.3333, 0.3333], and the default theta of the filter direction is +90 or -90, which happens to correspond to the image artifact caused by the partial volume effect.

## RESULTS AND DISCUSSION

**Figure 2:**
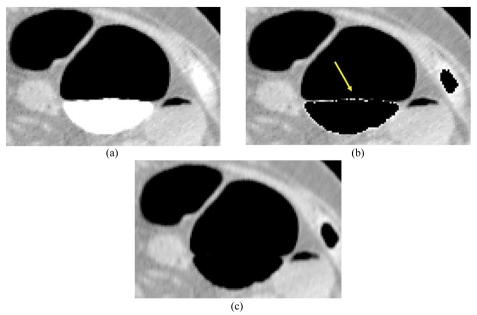
Result of electronic cleansing to an axial image of CT colonography. (Display condition is as follows: thresholding level 160HU, Window Center -200HU, and Window Width 650HU) (a) CTC before EC, (b) Result of segmentation by thresholding and (c) Result of EC using the authors' developed method.

The algorithm suggested in this study produces a smooth edge along the colon wall and reduces the artifact created by the by the boundary between the air lumen and the tagged regions (Figure 2). Simple thresholding segmentation will show a sharp image at the boundary, and aliasing effects appear at the inner colon wall (Figure 2(b)).

**Figure 3:**
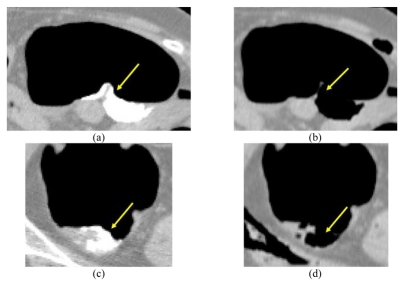
Different type of residual fecal debris with and without the authors' developed algorithm. (a) CTC before EC, (b) Result of EC, (c) CTC before EC, (d) Result of EC.

Figure 3 shows a cleansed and an uncleansed image, both obtained using the algorithm that has been developed here, and both with the same window level and window width (-150 and 700 respectively).

**Figure 4:**
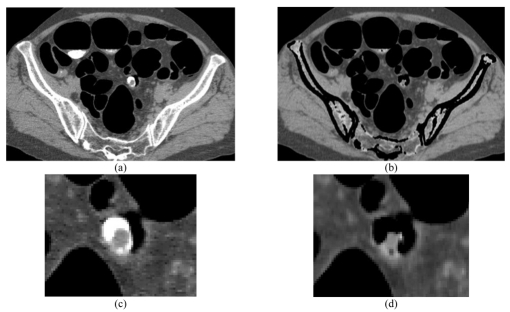
Result of EC to the colonic abnormality. (a) CTC before EC, (b) Result of EC, (c) Magnified image of (a) and (d) Magnified image of (c).

Cleansed images show fine soft tissue area without any trace of tagged material. True polyps however, are clearly illuminated on the CTC due to the barium tagging of stools adhering to the colon wall that increases their resolution [12]. The main flaw in solid-stool tagging is the diagnostic error possible with the barium coating of a true colonic abnormality. The process developed here for EC may not remove the entire layer of barium coating, even though it removes tagged materials adjacent to a lesion or other abnormality (Figure 4). Still, this algorithm showed a better quality image with a smoother boundary rather than the matrix convolution of the ‘magic squares’ segmentation technique [14].

Recently, 3D endoluminal rendering for polyp detection in CT colonography has become possible [15], and the optimal surface-rendering threshold value for accurate polyp measurement is approximately -500HU [16].

**Figure 5:**
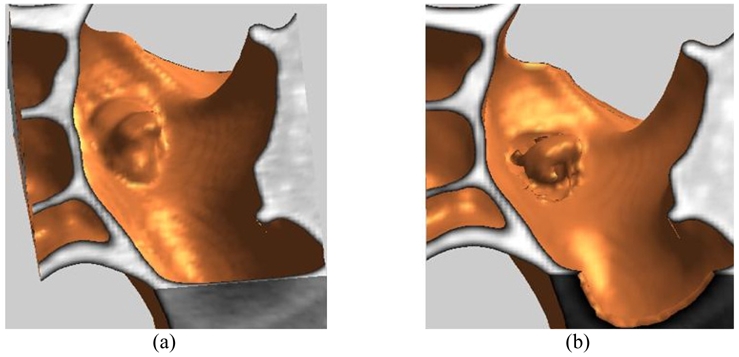
Three dimensional surface display between before and after EC processing. (Thresholding level is -500HU for the surface rendering) (a) Surface view of CTC before EC and (b) Surface view of CTC after EC.

Figure 5 shows the 3-dimensional displays before and after EC processing. While the thresholding level for surface rendering was set at -500 HU, the EC algorithm developed in this study removed only tagged materials and left the abnormal tissue in 3-D form. Compared to a 3-D structural algorithm like the eigenvalues of a 3-D Hessian matrix [17], the algorithm here is far more simple and has a faster processing speed for using 2-D filters in combination.

The only limitation of this developed algorithm is that it does not support a multi-directional filter in order to reduce the complex shape of partial volume defects. Also, due to the lack of uniform density variance inside the fecal residue, this algorithm failed to cleanse low-density residual fluid and uneven tagging residue. Clearly, the algorithm needs an improved processing speed along with maintaining accurate normal anatomical structure.

## CONCLUSIONS

It is suggested that simple image processing for electronic cleansing (EC) is based on both an air material template that reproduces the partial volume effect, and also the vertical motion filter. In the thresholding level for 3-D surface displays, the EC algorithm removed only tagged materials without removing the artifact. Accordingly, this approach may need further confirmation by subsequent research utilising a larger amount of stool tagging data in the CTC.
